# Three Dimensional Upper Limb Joint Kinetics of a Golf Swing with Measured Internal Grip Force

**DOI:** 10.3390/s20133672

**Published:** 2020-06-30

**Authors:** Hyeob Choi, Sukyung Park

**Affiliations:** Department of Mechanical Engineering, Korea Advanced Institute of Science and Technology (KAIST), Daejeon 34141, Korea; hyeobchoi@kaist.ac.kr

**Keywords:** internal grip force, three dimensional (3D), golf swing, upper limb, joint, kinetics

## Abstract

The biomechanics of a golf swing have been of interest to golfers, instructors, and biomechanists. In addition to the complexity of the three-dimensional (3D) dynamics of multi-segments of body, the closed-chain body posture as a result of both hands holding a club together makes it difficult to fully analyze the 3D kinetics of a golf swing. To identify the hand-grip joint force and torque applied by each hand, we directly measured the 3D internal grip force of nine registered professional golfers using an instrumented grip. A six-axis force-torque sensor was connected to a custom-made axially separated grip, which was then connected to a driver shaft using a manufactured screw thread. Subjects participated in two sessions of data collection featuring five driver swings with both a regular and customized sensor-embedded grip, respectively. Internal grip force measurement and upper limb kinematics were used to calculate the joint force and torque of the nine-linkage closed-chain of the upper limb and club using 3D inverse dynamics. Direct measurement of internal grip forces revealed a threefold greater right-hand torque application compared to the left hand, and counterforce by both hands was also found. The joint force and torque of the left arm tended to precede that of the right arm, the majority of which had peaks around the impact and showed a larger magnitude than that of the left arm. Due to the practical challenge of measuring internal force, heuristic estimation methods based on club kinematics showed fair approximation. Our results suggest that measuring the internal forces of the closed-chain posture could identify redundant joint kinetics and further propose a heuristic approximation.

## 1. Introduction

The biomechanics of a golf swing have been of interest to golfers, instructors, and biomechanists [1,2,3,4]. To quantitatively coach the appropriate golf swing posture, whole-body kinematics of professional golfers have been examined and compared with those of amateur golfers. Significant kinematic indices were reported between the pros and amateurs, and these differences were highlighted with subjects’ handicaps [5,6,7,8]. The rotational speed of the body segment showed sequential maximum peaks from the proximal to distal joints among skilled golfers [9,10]. As a cause of motion, identifying joint force and torque would help to understand the actuation strategy and to quantify their mechanical and physiological effects, such as impulse during impact, fatigue, and injury risks [11,12]. However, estimating joint kinetics obtained from inverse dynamics based on the kinematic measurement is challenging due to the complexity of the three-dimensional (3D) dynamics of multiple body segments. Moreover, with both hands holding a club together, the upper limbs make a closed-chain posture, and the inverse dynamics is not identifiable without first identifying the force and torque of one joint in the closed chain [13,14,15].

To resolve the closed-chain issue of joint kinetics analysis, studies employed a model simplification or equal distribution assumption. Previous studies have predicted optimal joint force and torque that results in maximum club speed using a 3D rigid body model [16,17]. To estimate the upper limb joint kinetics, 3D upper limb models for only the left arm were proposed, which resulted in the overestimation of the joint kinetics of the left arm. Nesbit estimated 3D whole body joint kinetics by assuming the equal contribution of each hand-grip joint force and torque, leaving the validation of assumption open [18]. Recently, Koike proposed to directly measure internal grip force using a 12-strain gauge instrumented club and used the measurement to estimate upper limb joint torque [13,19]. However, only three-axis partial components out of the full six-axis grip force and torque were measured from a single subject, which limited the validity of the reported joint torque and resulted in the generalization of the data.

To resolve the joint kinetics of the upper limb closed-chain during a golf swing, a six-axis force-torque sensor embedded club was developed and used to measure internal grip force and torque directly. Nine registered professional golfers participated in data collection by delivering driver swings with regular and customized sensor-embedded grip. Internal grip force measurement, along with the joint kinematics, were used to calculate the joint force and torque of the nine-linkage closed-chain system of the upper limbs and club by 3D inverse dynamics analysis. To our knowledge, this is the first study estimating the 3D joint kinetics of upper limbs during a golf swing by considering the internal grip force within the closed kinetic chain. Due to the practical challenge of directly measuring the internal grip force, we also proposed a heuristic estimation method for hand joint force based on club kinematics.

## 2. Methods

To resolve the issue of closed-chain joint kinetics of the upper limb during golf swing, the force and torque between the two club segments held by each hand (referred to as the “internal grip force” throughout the manuscript) were directly measured using a 6-axis force and torque sensor embedded at the customized grip. The measured internal grip force and the upper limb kinematic data were used to calculate the joint force and torque of the rest of the 9-linkage closed-chain system of upper limbs by 3D inverse dynamics analysis.

### 2.1. Design of the Six-Axis Internal Grip Force Measurement System

**Figure 1:**
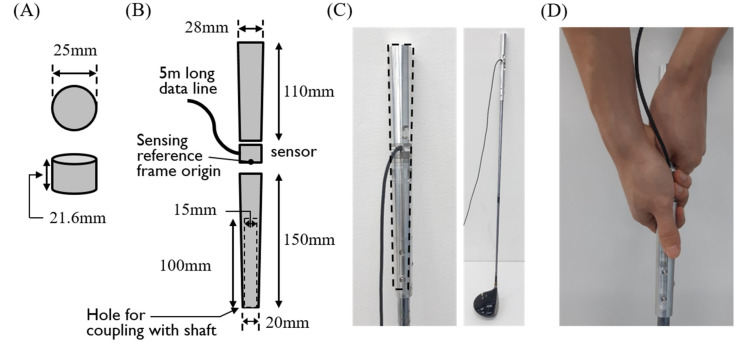
Sensor-embedded club. (**A**) Size of the sensor. (**B**) Drawing of the grip part. (**C**) Picture of the developed sensor-embedded club. The dotted line represents the size of a typical driver grip. (**D**) Picture of the hands and grip when the club is held.

The 6-axis force and torque measurement club was developed to measure the internal grip force and the force and torque between two club segments held by each hand (Figure 1). A 6-axis force-torque sensor (nano25, ATI, Apex, NC, USA) was screw connected around the middle of customized, two-pieces cylindrical grip (Figure 1A,B). The diameters of the bottom and the top of the grip were 28 mm and 20 mm, respectively, and the length was 280 mm, which are similar to the size of commercial driver grips while being large enough to align with the embedded force transducer straightly. The customized grip was connected to a driver club shaft by a manufactured screw thread. The cables of the transducer were organized to minimize the obstruction for subjects during data collection. The grip position of the club during testing was set to be as much like a regular golf club as possible while the subjects’ hands were slightly disturbed by transducer cables (Figure 1D). The swing trajectories with the customized and regular club grips were compared in the results.

### 2.2. Inverse Dynamics Model

**Figure 2:**
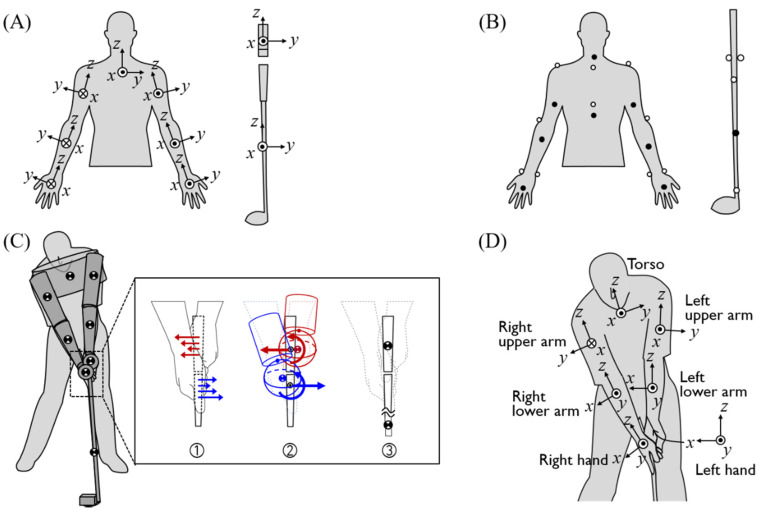
Three-dimensional upper limbs and club model for inverse dynamics analysis. (**A**) The local coordinate system of each segment. (**B**) Position of optical markers attached. White dots are markers attached to the front of the subject’s body, and black dots were attached to the back of the subject’s body. (**C**) The shape of the model. The inset shows the distribution of the force exerted on the club by both hands into the resultant force and torque in the inverse dynamics model, the shape of both hands, and the club segment. (**D**) The local coordinate system of each segment at the address posture.

The 3D joint force and torque of the upper limbs were calculated using inversed dynamics by measured segments kinematics and internal grip forces. A nine-segment rigid body model, including the torso and both upper arms, lower arms, hands, and two segments of the club, was used to demonstrate the dynamics of the upper limb during a swing (Figure 2). Each arm segment, hand, and the club were modeled as the frustum of cone, sphere, and the long hollow cylinder, respectively [20]. The clubhead of the driver was modeled as the hollow cuboid. The masses of the upper arm, lower arm, and hand were 0.028, 0.016, and 0.006 times the body mass, respectively [21], and the club was 551 g. All joints were modeled as ball joints. The rotational joint of the body was defined as the center of the end surface of the 3D frustum. The grip joint was the joint between two club segments and was defined as the origin of the sensing reference axis of the force-torque sensor imbedded in the club grip. The hand-grip joint, or hand joint, are used interchangeably throughout the manuscript and was determined as the center of the gripped club part (Figure 2C). A body-fixed local reference frame was used to describe the segment kinematics with the *z*-axis being the proximal direction of the length axis of the segment, and the y-axis being the lateral direction of the segment (Figure 2A,D and Table 1) [22]. The equation of motion for inverse dynamics analysis is as follows:

Equation of motion regarding translation

(1) $$m{\vec{a}}_{\mathrm{com}}=\Sigma {\vec{F}}_{\mathrm{ext}.}+m\vec{g}$$

Equation of motion regarding rotation

(2) $$\Sigma \left({\vec{T}}_{\mathrm{ext}.}+\vec{r}\times {\vec{F}}_{\mathrm{ext}.}\right)=I\dot{\vec{\omega }}+\vec{\omega }\times I\vec{\omega }$$

where *m*, *a*, *g*, *F*, *T*, *r*, *I*, and *w* are segment mass, acceleration of CoM (Center of Mass), gravitational acceleration, external force, external torque, moment arm, moment of inertia matrix, and angular velocity of the segment, respectively. The joint force and torque of the upper limbs were obtained by inverse dynamics analysis with an inward iteration.

When all the body segments are connected to more than two segments, thus forming a closed chain, a unique specification of the joint force and torque using inverse dynamics is not possible. Supposing that there is a simple closed-loop triangular posture model for both arms holding each other connected by a single clavicle, the kinematics of three segments are dependent on each other. The equations of motion for each segment are redundant, which caused the closed-chain kinetics to be underdetermined. You could easily establish this underdetermined closed-chain dynamics by varying the contact force and torque between the two hands while holding it together without changing joint kinematics. To resolve the issue of underdetermined closed-chain joint kinetics, additional joint force and torque information should be provided, which inspired us to develop a 6-axis internal grip force and torque measurement system.

### 2.3. Experiment

Nine healthy male professional golfers registered by the Korea Golf Federation or the Korea Professional Golf Association participated in the experiment. Average age, height, and weight were 37.8 ± 10.6 years, 82.0 ± 15.8 kg, and 174.0 ± 7.4 cm, respectively. All subjects were right-handed golfers. Before data collection, subjects signed the informed consent form approved by the Korea Advanced Institute of Science and Technology (KAIST) Institutional Review Board (KH2019-140). After about 5 min of warm-up swings, subjects participated in two sessions of data collection, which consisted of five full driver swing with a regular grip or sensor-embedded grip, respectively. The experiment with regular grip was always conducted first, and 5-min breaks were given between the two sessions. Subjects grasped the club with their usual grip method while locating the 6-axis force-torque sensor in the middle of both hands (Figure 1D).

The kinematics of the upper limbs and internal grip force and torque during swing were measured by an optical motion capture system (Hawk, Motion Analysis, Santa Rosa, CA, USA) and the custom-made force sensor-embedded club, respectively. Fourteen infrared cameras measured 24 markers attached to subjects’ acromioclavicular joint, back sides of the upper arms, lateral epicondyle of the humerus, back sides of the lower arms, the radius and ulnar styloid process, the head of the third metacarpal, the deepest point of incisura jugularis, processus xiphoideus, cervical vertebrae 7, thoracic vertebrae 10, top and middle of the club shaft, the neck of the clubhead, and the ball, respectively (Figure 2B) [22,23,24,25], at a 200 Hz sampling frequency. The force-torque sensors collected the 6-axis force and torque data with a sampling frequency of 400 Hz. All measurement data were synchronized by controlling the sample clock of the two systems through the Cortex motion capture software.

### 2.4. Data Processing

All collected data were filtered with a fifth-order bi-directional Butterworth low-pass filter with a cut-off frequency of 10 Hz. The position of the center of mass of each segment was estimated using the measured marker position and the developed upper limbs and club 3D model. The three-dimensional angular velocity of each segment was calculated using the orientation matrix of each segment model obtained from marker position information. The calculation method is as shown in Equation (3) and Equation (5) below. The velocity, acceleration, and angular acceleration of the segments were calculated through numerical differentiation [26]. Measured and calculated kinematic and kinetic data were then used for inverse dynamics. Inverse dynamics analysis is a method to calculate joint force and torque using motion information of body segments. In order to calculate the force and torque of a joint, it is necessary to know the motion information of one adjacent segment and all the forces and torques acting on the segment except for the joint force and torque. Therefore, there is an outward iteration method that analyzes from the ankle using the measured ground reaction force and an inward iteration method that analyzes from the hand that does not receive external force. We used an inward iteration method starting from the club toward the shoulder joints.

Angular velocity from the orientation matrix

$O_{i-1},\;O_{i+1}$: Orientation matrices of a segment at time $t_{i-1},\;t_{i+1}$

$R=O_{i+1}O_{i-1}^{-1}$: Rotation matrix representing segment rotation from $t_{i-1}$ to $t_{i+1}$

(3) $$\mathrm{Angle}\;\theta =\cos\left(\left(R_{11}+R_{22}+R_{33}-1\right)/2\right)$$

(4) $$\mathrm{Axis}\;p=O_{i-1}^{-1}*{\left[\begin{array}{ccc} \frac{R_{23}-R_{32}}{2\sin\theta } & \frac{R_{31}-R_{13}}{2\sin\theta } & \frac{R_{12}-R_{21}}{2\sin\theta } \end{array}\right]}^{T}$$

(5) $$\mathrm{Angular}\;\mathrm{velocity}\;v=\frac{\theta }{t_{i+1}-t_{i-1}}*p$$

**Figure 3:**
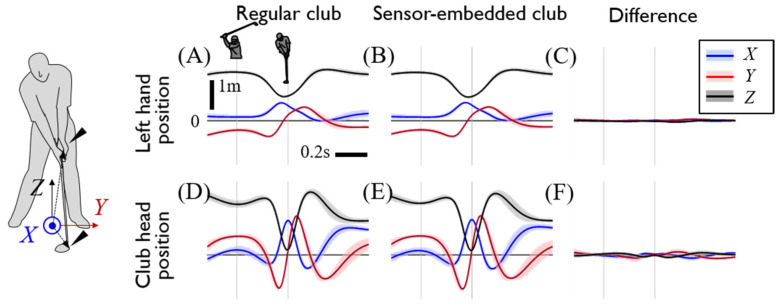
Swing motion data according to the used club type. Each row shows the position of the left hand (**A–C**) and clubhead (**D**–**F**). The first two columns show the motion data when the subject used a regular driver and the sensor-embedded driver, and the last column shows the difference. The blue, red, and black lines indicate the *x*, *y*, and *z* positions at the global coordinates, respectively. All motion data were the averages of driver swing data on all subjects. The two thin gray vertical lines indicate the backswing top and impact.

**Figure 4:**
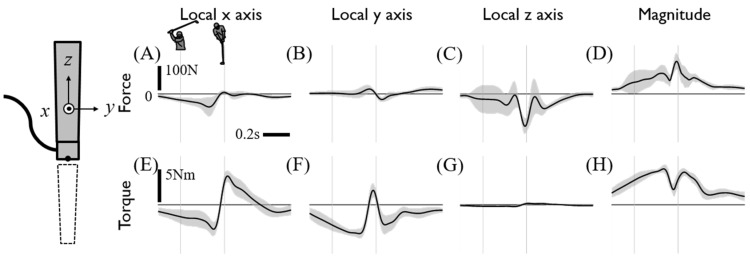
Internal grip force and torque. Each row shows force (**A**–**D**) and torque (**E**–**H**). Each column shows *x*, *y*, and, *z* values and the magnitude of force and torque at the club’s local coordinates. The force and torque act on the left-hand grip segment of the club at the sensing reference origin of the sensor and are averages of the driver swing data on all subjects. The two thin gray vertical lines indicate the backswing top and impact.

To verify whether the sensor-embedded club affected the subjects’ swing, the swing trajectories when using the regular club and sensor-embedded club were compared. The trajectory of the left hand and the clubhead and the main positions of the swing, showed relatively small difference with and RMS of 0.06 and 0.16 m, respectively (Figure 3). Through this, we concluded that the swing observed in the experiment with the sensor-embedded club could be regarded as a normal golf swing. Subjects also responded that the sensor-embedded club did not seem to affect their swing, although they felt some differences between the clubs, such as weight. The low-pass filtered internal grip force and torque measurements are presented in Figure 4. The force and torque reflect the interactive force and torque between the separated upper and lower grips, which are dominantly grasped by the right and left hand, respectively. With the reference frame of the lower part of the grip (Figure 4), the positive component of the x directional force implies pulling out a grip whereas the positive y and z directional forces imply pushing into it, and positive torque around x leads to the forward rotation of the club and positive y-directional torque leads to the downward rotation. Internal grip force measurement showed a pushing effect in the x-direction and a pulling force in the z-direction, respectively, during the downswing. With that, backward and upward torque were measured at the grip joint during the downswing phase followed by the abrupt reversal of their direction before an impact (Figure 4E,F). It implies that the upper part of the club received the opposite direction torque. The nine subjects showed consistent internal grip force and torque profiles, whereas the z directional joint forces showed large variations during the downswing phase.

**Figure 5:**
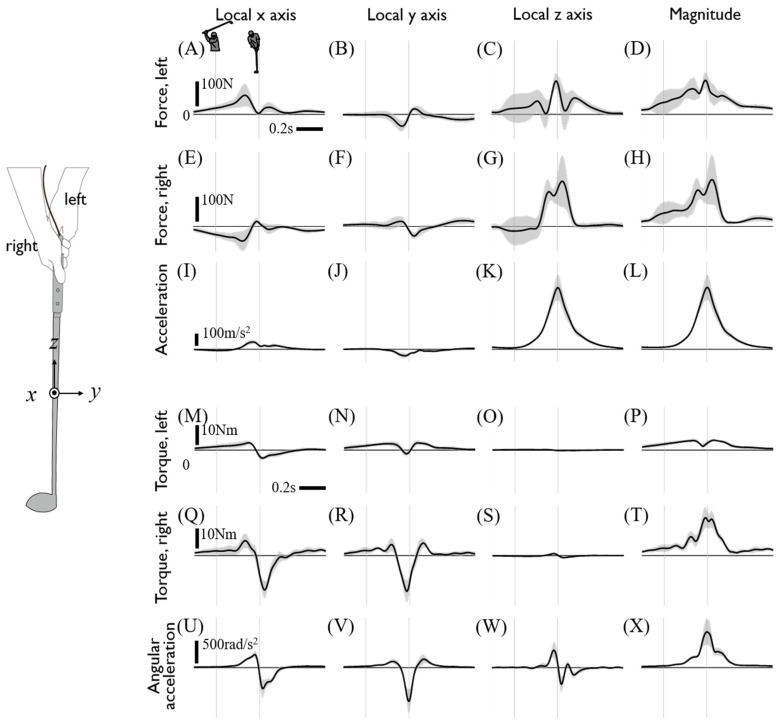
Hand-grip joint force and torque and kinematic data of the club. Each row shows the left (**A**–**D**) and right hand-grip joint force (**E**–**H**), acceleration of the club (**I**–**L**), left (**M**–**P**) and right hand-grip joint torque (**Q**–**T**), and the angular acceleration of the club (**U**–**X**), respectively. Each column shows the x, y, and z values and magnitude of each data at the club’s local coordinates. The force and torque act on the club, and all the data are the averages of driver swing data on all subjects. Two thin gray vertical lines indicate the backswing top and impact.

The direct measurement of the internal grip force made it possible to estimate left- and right-hand joint force and torque through inverse dynamics (Figure 5). To examine the relationship between the hand joint force/torque and the resultant linear/angular accelerations of the club, data are presented with local reference coordinates attached to the club, as shown in Figure 5. The hand-grip force of both hands in x- and y-directions showed approximatively opposite-directional profiles, making the inference of the grip forces from the resultant club acceleration difficult [13]. However, both the z-direction grip forces generate a large centripetal force in a similar manner and contribute to club rotation toward impact. The observations of x and y directional torque of both hands showed a similar trend to the corresponding angular acceleration of the club, with was about a threefold larger magnitude in right hand joint torque than that of the left hand (Figure 5M–R). Coherent to the angular motion of the club, positive x-direction torque was applied to generate radial acceleration followed by a rapid reversal to decelerate on impact. A sharp negative y-direction torque leads to re-cocking after the release of the club. Due to the z-direction moment of inertia with 1/200 of the other axes, the hand-grip torque around *z*-axis was negligible although the corresponding angular acceleration of the club showed significant torsional rotation.

**Figure 6:**
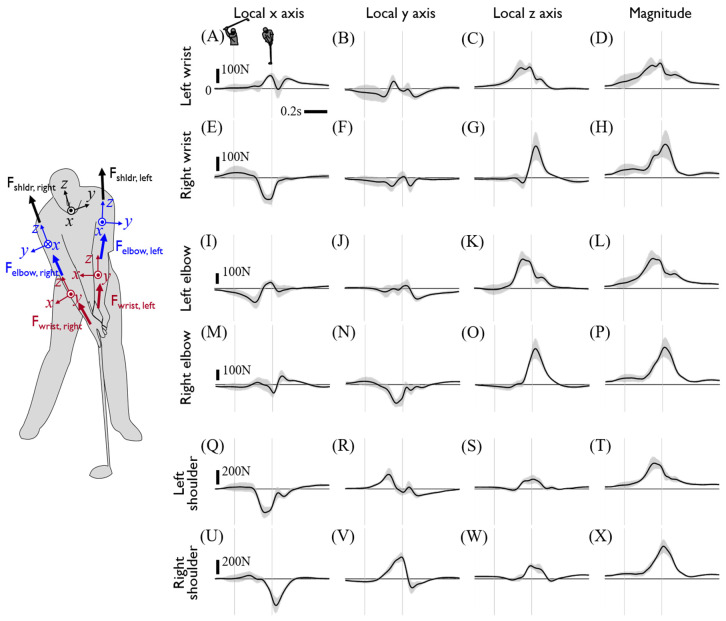
Calculated joint forces of the upper limbs. Each row shows the joint force of the left wrist (**A**–**D**), right wrist (**E**–**H**), left elbow (**I**–**L**), right elbow (**M**–**P**), left shoulder (**Q**–**T**), and right shoulder (**U**–**X**), respectively. Each column shows the x, y, and z values and the magnitude of joint force at the local coordinate of the proximal segment. The forces act on the distal segment of each joint and are averages of the driver swing data on all subjects. The two thin gray vertical lines indicate the backswing top and impact. The left silhouette shows the local coordinate of each segment at the address posture.

**Figure 7:**
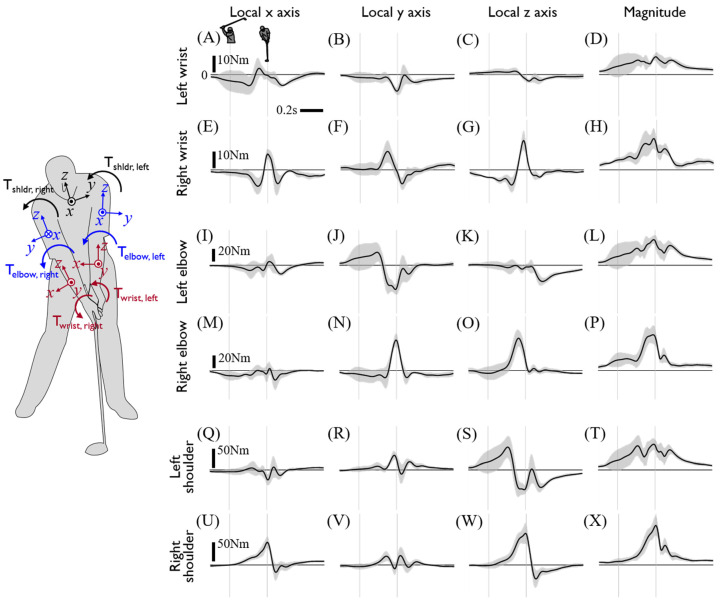
Calculated joint torques of the upper limbs. Each row shows the joint torque of the left wrist (**A**–**D**), right wrist (**E**–**H**), left elbow (**I**–**L**), right elbow (**M**–**P**), left shoulder (**Q**–**T**), and right shoulder (**U**–**X**), respectively. Each column shows the x, y, and z values and magnitude of joint torque at the local coordinate of the proximal segment. Torque acts on the distal segment of each joint and are the averages of driver swing data on all subjects. The two thin gray vertical lines indicate the backswing top and impact. The left silhouette shows the local coordinate of each segment at the address posture.

The upper limb joint kinetics calculated from inverse dynamics are presented in Figure 6 and Figure 7. The directions of joint kinetics are defined from the reference coordinates at the CoM of the proximal segment. Generally, joint force and torque of the left arm tends to precede that of the right arm as the majority of which have peaks around the impact and show a larger magnitude than that of the left arm. Specifically, either the onset or peak of the left arm joint force and torque precedes those of the right arm within a range of approximately 50–200 ms. The joint forces of both arms showed a similar trajectory with a slightly greater magnitude in the right arm. Whereas the x- and y-direction component of the wrist joint forces of each hand were oppositely directed, the rest of the joint force in both arms was applied in the same direction. Considering both arm segments are aligned with the radial direction of the clubhead’s rotational motion with respect to the center of the torso, the z-component of wrist and elbow joint force dominantly contributes to the centripetal force and showed peaks around the point of impact. A large joint torque of the right arm was observed in all three axes during mid-downswing to impact due to the drastic 3D postural change of the right arm (Figure 7U–W). Consistent with the observations of hand joint torque, right arm joint torque was also greater than the left arm’s, and this difference is highlighted in the distal joint.

## 3. Discussion

We developed a sensor-embedded club to measure internal grip force and torque directly and used the measurement to the upper limb joint kinetics during a golf swing. The estimated joint force and torque appeared to be coherent with the observed joint kinematics. To make a large swing arc at maximum speed at impact, both arms were straightened during the take away followed by torso rotation with right elbow flexion and wrist cocking toward the backswing top. During the downswing, the torso rotates back with the left shoulder extended, which is followed by the extension of the right elbow [27], which starts around mid-downswing. Joint kinetics estimation showed that during the downswing, the left limb joint kinetics tended to precede that of the right limb, which showed a maximum magnitude around impact (Figure 6 and Figure 7). Similarly, the left arm torque generally showed a monotonic increase starting from the downswing, whereas the right arm torque showed a rapid increase after mid-downswing with peaks around the moment of impact (Figure 7). Such joint kinetic profiles result in a rotational motion with the left shoulder extended followed by extension of the right elbow. The direct measurement of grip joint force revealed about a threefold greater right-hand torque application than that in the left hand and the existence of counterforce application by both hands (Figure 5). To our knowledge, this is the first study that quantitatively demonstrates the dominant role of right-hand grip torque in club rotation, which was consistently observed among all nine professional licensed golfers. Despite the negligible net hand-grip force in the x- and y-direction during the first half of the downswing, non-negligible hand-grip forces of similar magnitude and opposite directions were applied by each hand (Figure 5A–J). Considering the distance between the point of action in each hand-grip force, the counteracting joint forces in x- and y-direction contribute to the generation of the y- and x-direction angular acceleration of the club, respectively (Figure 2C), which could rectify a possible overestimation of the hand joint torque estimated purely from club rotation without measuring internal grip force. Our findings regarding the counteracting joint forces are consistent with the previous report that partially measured the hand-grip force of one professional golfer using a strain-gauge instrumented club [13], which may have attributed to slight quantitative discrepancies in the hand-grip force. Consistent with previous observations on sequential body segment rotation from the proximal to distal joint starting from the torso [9], we observed the sequential peaks of axial joint torque in the z-direction (Figure 7E,O). When swinging, the internal rotation of the upper arm, the extension of the elbow, and the internal rotation of the lower arm occur sequentially in the right arm. At this time, the torque generated as a result of the internal rotation of the upper arm is the Tz of the elbow, and the torque generated as a result of the internal rotation of the lower arm is the wrist Tz. The sequential peaks of joint torque in the z-direction are associated with this sequential movement.

**Figure 8:**
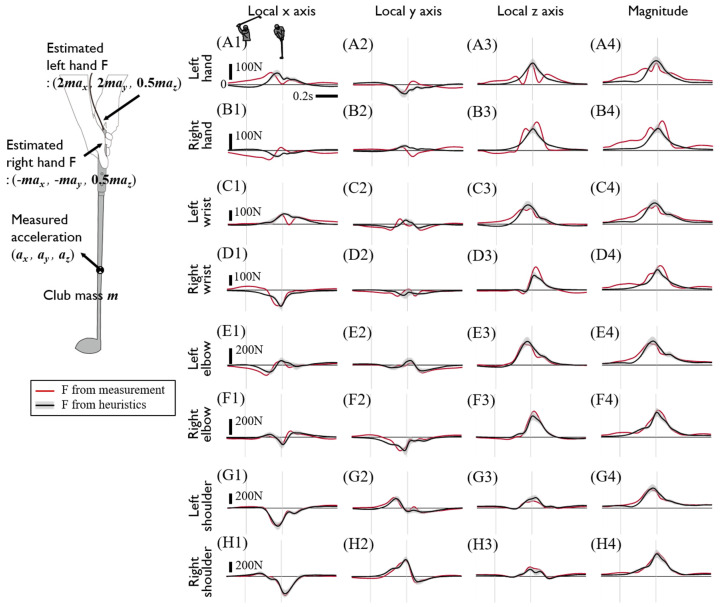
Calculated joint forces of the upper limbs using the measured internal grip force and torque or estimated hand-grip joint force and torque. Each row shows the joint forces of the left hand-grip joint (**A1**–**A4**), right hand-grip joint (**B1**–**B4**), left wrist (**C1**–**C4**), right wrist (**D1**–**D4**), left elbow (**E1**–**E4**), right elbow (**F1**–**F4**), left shoulder (**G1**–**G4**), and right shoulder (**H1**–**H4**), respectively. Each column shows the x, y, and, z values and the magnitude of joint force at the local coordinate of the proximal segment except for the hand-grip joint. The hand-grip joint force was analyzed using the club’s local coordinates. The black line with gray shading shows the joint force calculated from the estimated hand-grip joint force and torque, and the red line shows the joint force calculated from the measured internal grip force and torque. The forces act on the distal segment of each joint and are the averages of driver swing data pm all subjects. The two thin gray vertical lines indicate the backswing top and impact. The left picture briefly shows how to estimate the hand-grip joint force.

**Figure 9:**
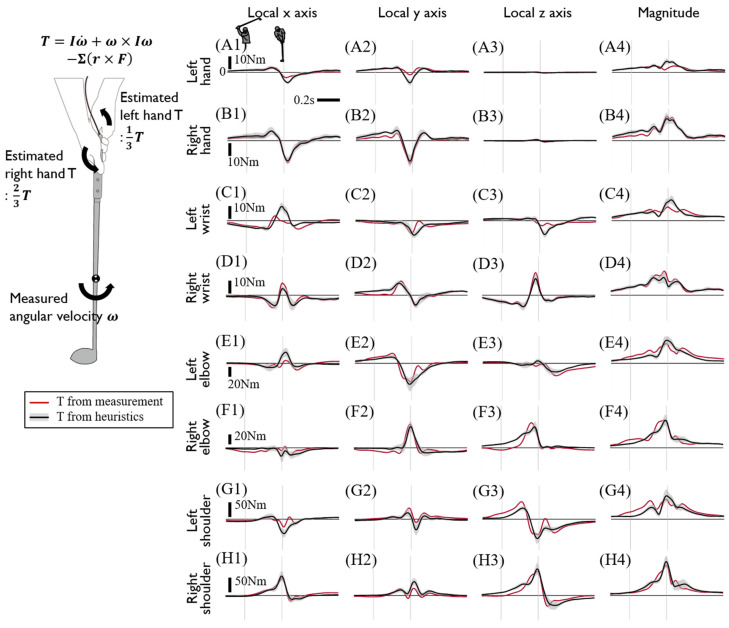
Calculated joint torques of the upper limbs using measured internal grip force and torque or estimated hand-grip joint force and torque. Each row shows the joint torques of the left hand-grip joint (**A1**–**A4**), right hand-grip joint (**B1**–**B4**), left wrist (**C1**–**C4**), right wrist (**D1**–**D4**), left elbow (**E1**–**E4**), right elbow (**F1**–**F4**), left shoulder (**G1**–**G4**), and right shoulder (**H1**–**H4**), respectively. Each column shows the x, y, and, z values and magnitude of joint torque at the local coordinate of the proximal segment, except for hand-grip joint. The hand-grip joint torque was analyzed using the club’s local coordinates. The black line with gray shading shows the joint torque calculated from the estimated hand-grip joint force and torque, and the red line shows the joint torque calculated from the measured internal grip force and torque. The torque acts on the distal segment of each joint and are the averages of driver swing data on all subjects. The two thin gray vertical lines indicate the backswing top and impact. The left picture briefly shows how to estimate the hand-grip joint torque.

Although the direct measurement of internal grip force and torque is necessary to identify upper limb joint kinetics, its measurement is more challenging than the measurement of club kinematics, which is often performed by a simple IMU (Inertial Measurement Unit) [28,29,30,31,32] or a motion capture system [33,34,35]. After observing the relatively small inter-subject variability in most of the six-axis grip sensor data, we then examined whether the hand-grip joint kinetics could be heuristically estimated from club kinematics. As such, we proposed an estimation method to determine hand-grip joint force and torque based on three heuristic tunings. First, to estimate the magnitude of the counteracting hand joint force by each hand, the weighted offset was added to the corresponding linear acceleration component of the club. The x- and y-directional left hand joint forces were estimated to be twice the club mass times the x and y acceleration of club, respectively, and the right hand joint force was determined by that the sum of both hand joint forces equal to the total force that the club receives. Second, the z-direction hand joint force, which is attributed to the centripetal acceleration of the club, was estimated to be equally divided by each hand. Finally, the observed dominance of the right arm torque was used as the weighted division of hand joint torque by left and right hand as 1 to 2. Despite this simple heuristic assumption, the estimated grip joint kinetics were reasonably well approximated to the data. (Figure 8 and Figure 9). It is worth noting that the effects of hand joint force errors on inverse dynamics decrease over proximal joint estimation due to the increased inertia (Figure 8). The mass of the upper and lower arm segment is about four and threefold heavier than the club mass, and this results in a greater magnitude of joint force and torque compared to the hand-grip joint. Therefore, the relative significance of erroneous hand joint force estimations when superposed with the force and torque of the elbow and shoulder joints would be reduced at the proximal joint.

**Figure 10:**
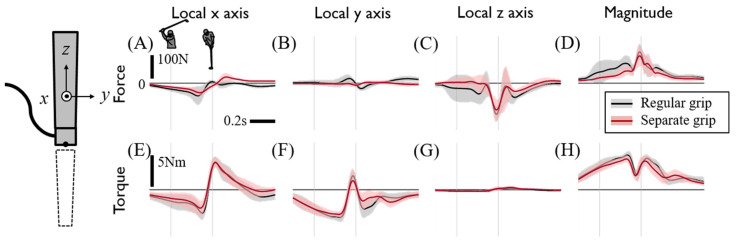
Internal grip force and torque under regular and separate grip posture conditions. Each row shows the force (**A**–**D**) and torque (**E**–**H**). Each column shows x, y, and z values and the magnitude of force and torque at the club’s local coordinates. The black line shows the internal grip force under regular grip posture conditions, and the red line shows the internal grip force under separate grip posture conditions. The forces and torques act on the left-hand grip at the sensing reference origin of the sensor and are the averages of driver swing data on all subjects. The two thin gray vertical lines indicate the backswing top and impact.

Although the estimation of 3D upper arm joint kinetics was firstly presented by directly measuring the internal grip force, possible causes of estimation errors due to instrumentation set up and model simplification should be stated. First, the increased weight of the instrumented grip (~350 g) compared to a regular driver grip (~100 g) would increase the total magnitude of the joint force and torque proportional to the weight change. If we assume that the weight distribution of the instrumented grip is approximately uniform and the center of mass would not change much, then the increased grip mass would affect the estimation of the hand joint force/torque in a similar manner and would not significantly change the main finding of this study. Secondly, the hand grip posture by covering the left thumb by thenar eminence of the right hand leads to the coupling of the grip forces of both hands. Then, the embedded force sensor measurement could possibly underestimate the difference in the hand-grip forces. To examine this overlap effect on grip force measurement, subjects also performed swings by grasping the club with two hands completely separated. Although subjects reported unfamiliarity when swinging with a separated grip, the measured internal grip forces were not significantly different (Figure 10), implying that hand force coupling would not change the main claim of this study either. Thirdly, for safety purposes, the experiments were performed with sponge golf balls made of polyurethane foam, which would lead to an underestimation of the impulse of impact, especially the y-directional impulse [36]. Finally, the body segment was modeled as a rigid body, which would not allow the torsional motion. However, the human body segment showed a twisting motion during the swing, which would result in the overestimation of joint torque. Considering the twisting behavior of the forearm, the effective moment of inertia around its axial axis would be reduced roughly by half, which would lead to the proportional reduction of joint torque as well.

## 4. Conclusions

We analyzed the 3D upper limb joint kinetics of a golf swing by measuring the internal force of the upper limb closed-chain through the development of a sensor embedded club. We confirmed that there are kinetic properties, such as counteracting hand joint forces that can only be observed through the measurement of internal force. This implies that the internal force should be measured for the kinetic analysis of movements in which the human body forms a closed chain. However, for golf, we proposed a method to roughly estimate the internal force from the club’s kinematics information, and we expect that the method would be helpful in future golf kinetics studies.

## Figures and Tables

**Table 1:** Local axes of each segment.

Segment	Local *x*-Axis	Local *y*-Axis	Local *z*-Axis
Both club segment	Cross product of the local *y*- and *z*-axis	The direction of the clubface	Grip direction of the longitudinal axis
Hand	Perpendicular –direction to the palm (left) and back of hand (right)	Cross product of local *z*- and *x*-axis	The Proximal direction of longitudinal axis
Lower arm	Cross product of the local *y*- and *z*-axis	Lateral direction of wrist flexion axis	``
Upper arm	``	Lateral direction of elbow flexion axis	``
Torso	``	Right shoulder to left shoulder direction	Upper direction of longitudinal axis

`` means that the content is the same as the content in the upper cell.
